# Practices in Wound Healing Studies of Plants

**DOI:** 10.1155/2011/438056

**Published:** 2011-05-26

**Authors:** Rupesh Thakur, Nitika Jain, Raghvendra Pathak, Sardul Singh Sandhu

**Affiliations:** ^1^Biochemical Research Laboratory, Centre for Scientific Research and Development, People's Group, Bhopal, Madhya Pradesh 462037, India; ^2^Department of Biotechnology, Maharishi Markandeshwar University, Mullana, Ambala, Haryana 133207, India

## Abstract

Wounds are the result of injuries to the skin that disrupt the other soft tissue. Healing of a wound is a complex and protracted process of tissue repair and remodeling in response to injury. Various plant products have been used in treatment of wounds over the years. Wound healing herbal extracts promote blood clotting, fight infection, and accelerate the healing of wounds. Phytoconstituents derived from plants need to be identified and screened for antimicrobial activity for management of wounds. The *in vitro* assays are useful, quick, and relatively inexpensive. Small animals provide a multitude of model choices for various human wound conditions. The study must be conducted after obtaining approval of the Ethics Committee and according to the guidelines for care and use of animals. The prepared formulations of herbal extract can be evaluated by various physicopharmaceutical parameters. The wound healing efficacies of various herbal extracts have been evaluated in excision, incision, dead space, and burn wound models. *In vitro* and *in vivo* assays are stepping stones to well-controlled clinical trials of herbal extracts.

## 1. Introduction

Optimum healing of a cutaneous wound requires a well-orchestrated integration of the complex biological and molecular events of cell migration and proliferation and of extracellular matrix deposition and remodeling. Wound care and maintenance involves a number of measures including dressing and administration of painkillers, use of anti-inflammatory agents, topical systemic antimicrobial agents, and healing promoting drugs. Burn wound care is needed according to the severity of burn. The aim of wound care is to promote wound healing in the shortest time possible with minimal pain, discomfort, and scarring to the patient and must occur in a physiological environment, conducive to tissue repair and regeneration. This systematic review of *in vitro* and *in vivo* experiments could promote closer collaboration between the research communities and encourage an iterative approach to improving the relevance of various models to clinical trial design.

## 2. Wound

Wound is defined as disruption of cellular, anatomical, and functional continuity of a living tissue. It may be produced by physical, chemical, thermal, microbial, or immunological insult to the tissue. When skin is torn, cut, or punctured it is termed as an open wound and when blunt force trauma causes a contusion, it is called closed wound, whereas the burn wounds are caused by fire, heat, radiation, chemicals, electricity, or sunlight [1, 2].

## 3. Wound Healing

Wound healing is the interaction of a complex cascade of cellular and biochemical actions leading to the restoration of structural and functional integrity with regain of strength of injured tissues. It involves continuous cell-cell interaction and cell-matrix interactions that allow the process to proceed in different overlapping phases and processes including inflammation, wound contraction, reepithelialization, tissue remodelling, and formation of granulation tissue with angiogenesis. The phases of wound healing normally progress in a predictable, timely manner, and if they do not, healing may progress inappropriately to either a chronic wound such as a venous ulcer or pathological scarring such as a keloid scar [3].

## 4. Role of Plants

Plants have the immense potential for the management and treatment of wounds. A large number of plants are used by tribal and folklore in many countries for the treatment of wounds and burns. These natural agents induce healing and regeneration of the lost tissue by multiple mechanisms. These phytomedicine are not only cheap and affordable but are also safe. The presence of various life-sustaining constituents in plants has urged scientist to examine these plants with a view to determine potential wound healing properties [4]. Many phytopharmaceutical laboratories are now concentrating their efforts to identify the active constituents and modes of action of various medicinal plants [5]. The medicinal value of these plants lies in bioactive phytochemical constituents that produce definite physiological action on the human body [6]. These constituents include various chemical families like alkaloids, essential oils, flavonoids, tannins, terpenoids, saponins, and phenolic compounds [7]. 

**Figure 1:**
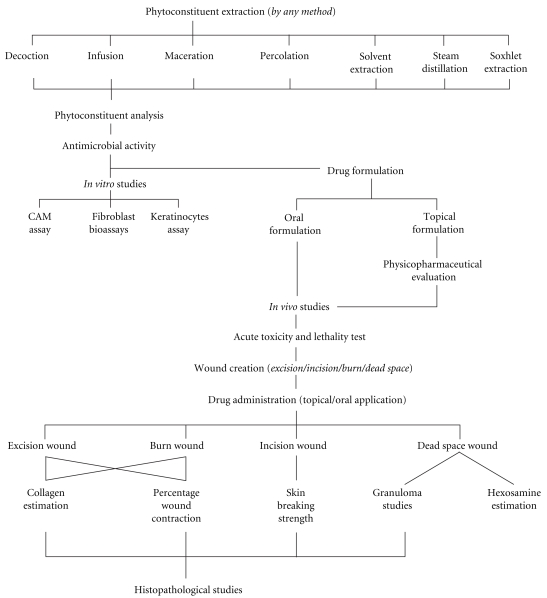
Schematic illustration of practices in wound healing studies of plants.

The screening of herbal extracts has been of great interest to the scientists for the discovery of new effective drugs [8]. A number of reports concerning the antibacterial, anti-inflammatory, and wound healing activity of various plants have appeared in the literature, but the vast majority has yet to be explored. Various pharmacological reports are available on plants employing different wound healing models and its underlying molecular mechanism for the validation of their traditional claims and development of safe and effective and globally accepted herbal drugs for wounds. The schematic illustration of practices in wound healing studies of plants is shown in Figure 1.

## 5. Phytoconstituent Extraction

**Figure 2:**
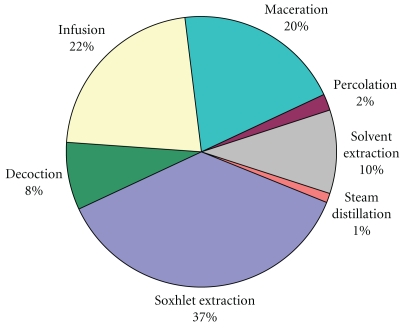
Percentage utilization of different methods of phytoconstituent extraction, calculated from the published research articles.

It involves the separation of the medicinally active portion from plant using selective solvents. Standard techniques separate out soluble metabolites leaving behind insoluble cellular marc. The products so obtained are relatively complex mixtures of a number of groups of metabolites either in liquid form or semisolid state or after removing the solvent resulting in dried powdered extracts intended for oral or topical use. These include classes of preparations known as decoctions, infusions, fluidextracts, tinctures, pilular extracts, or powdered extracts. The herbal extract thus obtained may be ready for use as a medicinal agent as such, or it may be further processed to be incorporated in any dosage form. An extract may be further processed through various techniques of fractionation to isolate individual chemical entities to be used as modern drugs [9]. There are many processes patented throughout the world for extraction of plant ingredients. Percentage utilization of different methods of phytoconstituent extraction is presented in Figure 2. The data was calculated from the published original research articles compiled from various sources during the composition of this paper. The general techniques employed for extraction of phytoconstituents are as follows.

### 5.1. Decoction

In this process, the plant material is boiled in a specified volume of water for a defined time; it is then cooled and strained or filtered. This procedure is suitable for extracting water-soluble, heat-stable constituents. The starting ratio of plant material to water is fixed, ranging from 1 : 4 or 1 : 20. The volume is then brought down to one-fourth its original volume by boiling during the extraction procedure. Then, the concentrated herbal extract is filtered and used as such or processed further [10].

### 5.2. Infusion

It is prepared by leaving the plant material soaked in the solvent (generally at room temperature) for a period of time, with or without intermittent shaking, followed by filtration to separate away the plant debris. If the plant material has settled, then the upper solvent extract can be decanted off and replaced if necessary with fresh solvent. These are dilute solutions of the readily soluble constituents of plant material [11].

### 5.3. Maceration

It is a process of preparation of a herbal extract by soaking plant material in water, vegetable oil, or some organic solvent. The whole or coarsely powdered, air-dried, and pulverised plant material is placed in a stoppered container with the solvent and allowed to stand at room temperature for a period of at least 3 days with frequent agitation until the soluble matter has dissolved. The mixture then is strained, the marc (the damp solid material) is pressed, and the combined liquids are clarified by filtration or decantation after standing [12].

### 5.4. Percolation

The plant material is packed into the column with a tap at the lower end, and an intermediary filter or sinter is provided to prevent escape of the solid material. The tap is opened and the extraction solvent (at room temperature or above) is poured in at the top and allowed to trickle through the material. Chemicals which are extracted are collected in a suitable container. The evaporation results in the dry herbal extract. The process can be repeated as many times as necessary to ensure full extraction [13].

### 5.5. Solvent Extraction

When the plant material comes in contact with a solvent, the soluble components in the material move to the solvent which results in the mass transfer of soluble active ingredient to the solvent, and this takes place in a concentration gradient. The rate of mass transfer decreases as the concentration of active ingredient in the solvent increases, until equilibrium is reached; that is, the concentrations of active ingredient in the plant material and the solvent are the same. Since mass transfer of the active ingredient also depends on its solubility in the solvent, heating the solvent can enhances the mass transfer. Moreover, if the solvent in equilibrium with the plant material is replaced with fresh solvent, the concentration gradient is changed [14].

### 5.6. Steam Distillation

This is carried out for the extraction of essential oils by mixing the plant material with water and heated to boiling. The emergent vapours are collected and allowed to condense, and the oil separates from the water. However, if prolonged boiling is to be avoided, then the steam from a separate generator can be passed either through plant material suspended in water but not boiled (hydro steam distillation) or directly through the plant material laid out on a mesh arrangement between the steam inlet and the condenser (direct steam distillation). Once the oil and steam have condensed, the two layers may be separated by physical means using separating funnel [15].

### 5.7. Soxhlet Extraction

The plant material is placed inside a thimble made from thick filter paper, which is loaded into the main chamber of the Soxhlet extractor. This extractor is placed onto a distillation flask containing the solvent. The Soxhlet is then equipped with a condenser, and the solvent is heated to reflux. The warm solvent vapour travels up a distillation arm and floods into the chamber housing the thimble. When the chamber is almost full, it gets automatically emptied by a siphon side arm back down to the distillation flask. This cycle may be allowed to repeat many times so that the desired compound gets concentrated in the distillation flask [2, 16].

The solvent extracts are filtered, concentrated under reduced pressure (30 ± 10 mbar) in a rotary evaporator at 30°C–60°C to a syrupy consistency and finally dried in vacuum desiccator using sodium sulphide [2]. Whereas the aqueous extracts are collected and filtered, the concentrated material is reduced to a mass at room temperature, and water is removed by placing it in desiccators and then submitted to lyophilization by a freeze-dryer, to produce powdered form of the extract. Lyophilization removes the water and stabilises the extract so that it can retain satisfactory pharmacological activity during long term storage [17]. The weight of the dried mass is recorded and used for experimental studies.

## 6. Phytoconstituent Analysis

The presence of alkaloids is detected by Dragenddorff's reagent, Mayer's reagent, Hager's reagent, and Wagner's reagent whereas steroids and triterpenes by Salkowski's and Liberman Burchardt's tests and glycosides by Legal's test, Borntrager's test while carbohydrates by Molisch's test, Fehling's test, Barfoed's test, and Benedict's test. Proteins and amino acids are distinguished by Millon's reagent and Biuret's test. Flavonoids are identified by alkaline reagent test, lead acetate test, Shinoda test, and zinc hydrochloric acid reduction test and saponins by froth test and foam test. Phenolic compounds and tannins are spotted by ferric chloride test and lead acetate test whereas tannins by the gelatin test [18, 19].

## 7. Antimicrobial Activity

For the disc diffusion assay, 5 mm discs of Whatman filter paper (no. 1) is immersed in the slurry of herbal extract (mg/mL) made in sterile water. The discs are then placed onto the surface of nutrient agar plates inoculated with 24 h old culture of the appropriate bacteria and scored for the presence of a zone of clearing around the disc after 48 h of incubation at 37°C. Control disks contained solvents, whereas commercial drugs are used as standards [11].

By agar well diffusion method, sterilized Muller Hinton agar plates are seeded with 1 × 10^6^ cfu/mL concentration of test organisms. Using a sterile cork borer (6-7 mm diameter), wells are bored on the surface of agar and the solution of herbal extract (mg/mL) in dimethylsulfoxide (DMSO) is introduced into appropriate well. Blank DMSO in separate well serves as control. The plates are kept undisturbed at room temperature for prediffusion period of 30 min and incubated for 24 h at 37°C. After incubation, the diameter of the inhibition zone for each well is measured and the mean obtained [20]. 

The minimum inhibitory concentration (MIC) is determined using the agar dilution technique. Serial concentrations of the herbal extract (mg/mL) are incorporated into nutrient agar plates. Thereafter, 24 h actively growing culture of the test organism is then streaked on the plate. MIC for each organism is taken as the lowest concentration of the herbal extract in the nutrient agar that inhibited the visible growth of the organism after 24 h of incubation at 37°C [21].

## 8. *In vitro* Studies

**Figure 3:**
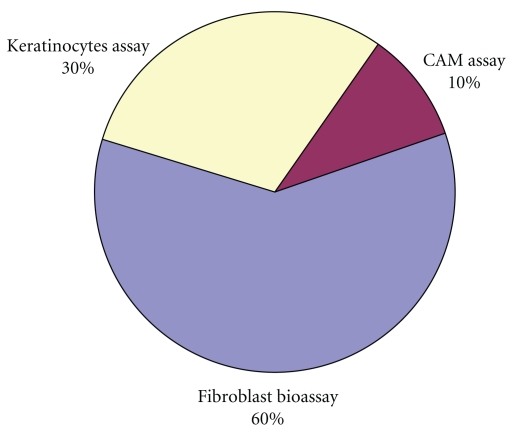
Percentage utilization of different techniques for *in vitro* studies, calculated from the published research articles.

Tissue repair concerns many events, both contractile and chemical, and the causing of a wound on any body surface stimulates the wound healing in the skin, which is a complex process characterized by angiogenesis reepithelialization, granulation tissue formation, and remodeling of extracellular matrix. These steps, accomplished primarily by dermal fibroblasts and keratinocytes, are well orchestrated by bioactive molecules including growth factors, cytokines and their receptors, and matrix molecules. The main cell architect participating in this process of healing by contraction is the fibroblast, which is also involved in the synthesis and deposition of the extracellular matrix. Hence, the fibroblast *in vitro* model is integral to correlating the contractile events of wound healing [22]. Therefore, the human dermal fibroblast (HDFs) growth-stimulating activity of plant compounds is an appropriate technique by which to determine the wound healing properties of the test compounds [23]. Key to wound healing processes are the proliferation, migration, and functioning of fibroblasts and keratinocytes, thus they are the basis of *in vitro* studies. Percentage utilization of different techniques for *in vitro* studies is presented in Figure 3. These *in vitro* assays can be useful, since they are quick, relatively inexpensive, and can be used to screen a wide variety of conditions or samples simultaneously but are incapable of replicating all the factors involved in complex processes of wound healing.

### 8.1. Chick Chorioallantoic Membrane (CAM) Assay

The CAM model is used to assess the angiogenic activity of herbal extract. Nine-day-old fertilized chick eggs are selected, and a small window of 1.0 cm^2^ is made in the shell. The window is opened, and a sterile disc of methylcellulose loaded with herbal extract is placed at the junction of two large vessels on CAM. The window is resealed by tape, and the eggs are incubated at 37°C in a well-humidified chamber for 72 h. Then, eggs are opened, and new blood vessel formation is observed in CAM treated by herbal extract which are compared with CAM containing disc without herbal extract (control) and the CAM treated with 10 *μ*L 1000 AU/mL bFGF (Fibroblast Growth Factor) as a standard [24]. The photographic images of the CAM model are analyzed for quantitative morphometric analysis of the density of blood capillaries in terms of the number of red pixel per unit areas using ImageJ software and AngioQuant software [25, 26].

### 8.2. Fibroblast Bioassay

Human dermal fibroblast (HDF) cells from postauricular surgery are grown to confluence after which they are removed from the culture flask using trypsin/EDTA after washing with phosphate buffer saline (PBS). HDFs are resuspended in 50 mL of Dulbeccos' modified eagle medium (DMEM), centrifuged at 2600 rpm for 5 min, and seeded in a 96-well sterile microtitre plate at a density of 11 × 103 cells/well in DMEM containing 10% fetal calf serum (FCS), 0.02% fungizone, 1% penicillin, and 2% streptomycin. After 24 h, the media is removed by aspiration. Solutions are initially solubilized in water and diluted in DMEM containing 0.5% fetal bovine serum (FBS) (0.5% FBS is the maintenance level required for HDF growth) to give a final concentration of 100 *μ*g/*μ*L. Solutions are filtered through a 0.2 *μ*M sterile filter prior to addition to the cells, and 1 : 1 serial dilutions are prepared. Aliquot (200 *μ*L) of herbal extract, in triplicate, is added to each well. The plates are left to incubate for 3 d. The neutral red assay is used to analyze the effects of the herbal extract on the growth of fibroblasts. Neutral red dye (1.2 mL) is added to 78.8 mL of Hanks' balanced salt solution (HBSS). This is incubated for 10 min at 37°C after which it is centrifuged at 2600 rpm for 5 min and 100 mL added to each well. The plates are incubated for 2.5 h, the media is tipped off, and the cells were washed with 100 *μ*L of 1% formic acid followed by 100 *μ*L of 1% acetic acid. The absorbance is recorded at 550 nm, and the values obtained for the solutions are compared with the control (0.5% FCS) [23].

The fibroblasts can also be cultured in a laboratory from fetal rat skin and subcultured through three passages before use. Cells are incubated in an atmosphere of 5% CO_2_, 95% air at 37°C in a tissue culture incubator, and suspended at 0.5 ×10^5^ viable cells/5 mL in the growth media (DMEM). The viability of the cells is assessed by trypan blue exclusion. The suspended cells are fortified with 10% FCS and allowed to equilibrate for 3 d. Herbal extract and standard commercial drug as control is introduced into the medium separately on day 3 at a dose of 40 mL in a concentration of 1 mg/mL, in phosphate buffered saline pH 7.4. The medium is changed periodically. On day 9, the medium is aspirated, and cells are washed with phosphate buffered saline. Half the quantity of cells is assayed for hydroxyproline content and the other half for DNA. Values are expressed as mg hydroxyproline/100 mg DNA [22].

### 8.3. Keratinocytes Assay

Keratinocytes can be isolated from the residual skin samples removed during surgery or human foreskins that can be obtained from circumcised newborn babies. The residual skin graft is treated overnight with 0.3% solution of trypsin at 4°C, whereas foreskins are washed extensively with multiple changes of PBS, subcutaneous tissue is removed, and the remaining samples are enzymatically dissociated in multiple changes of 0.25% trypsin and versene (50 : 50). Epidermal sheets are peeled from the dermis, minced, and dispersed in trypsin solution by repeated pipetting. The cell suspensions are pelleted from the trypsin solution, sequentially suspended, and washed with PBS by centrifugation at 1000 × g for 5 min at 20°C. Prior to cocultivation with keratinocyte, proliferation activity of fibroblasts is stopped using a solution of Mitomycin C at a concentration of 25 *μ*g/mL for 3 h. These cells are raised in a tissue culture dish with feeder cells (J2 mouse fibroblasts). Feeder cells are seeded on a cover glass at a density of 25,000 cells/cm^2^ and cultured for 24 h. A suspension of keratinocytes (20,000 cells/cm^2^) is then added, and cells are cultivated in a keratinocyte serum-free growth medium (KGM) at 37°C and 3.3% CO_2_. Cultures are maintained in a growth medium consisting of DMEM and Ham's nutrient mixture F12 at a 3 : 1 ratio. Cultures are fed every 3 days and subcultured by dispersal in 0.025% trypsin in PBS and replated at a split ratio of 1 : 3. Cultures are used between passages 2 and 3 [27, 28].

For *in vitro* assay human keratinocytes, either second or third passage (P2 or P3) is trypsinized, seeded into 32 mm, tissue culture dishes at densities from 5 × 10^5^ to 8 × 10^5^ cells/dish in KGM, and fed every 2 d until they reached 100% confluence. The medium is then replaced with RPMI 1640/10% FCS. After 4 h a scratch is made with a micropipette tip, and cells are washed with PBS in order to remove loosened debris. RPMI 1640/2% FCS medium with or without the herbal extract (1 mg/mL to 300 mg/mL) is added to sets of 2 dishes per dose. Each dish is orientated on the bottom of a six-well plate lid and fixed using glue. For each scratch, four consecutive fields are selected using the following criteria: relatively little cell debris within the scratch, even scratch, with straight edges, both edges visible under a single field using the X 10 objective, fields not too close to either end of the scratch, where distortions uncharacteristic of the main part of the scratch could be seen to occur during the ‘‘healing” process. The coordinates on the vernier scales of the inverted microscope stage are noted for every field. The condition of each field is recorded on video at various intervals over periods up to 72 h, as long as there was still a denuded area. Photographs of each field are also taken at the same time points. The average percentage of the initial area still denuded is calculated for each treatment set for the total of 8 fields per set [29].

To study the effect of plant extract on keratinocyte migration, the normal human keratinocytes are cultured to confluence in a six-well culture plate, and the culture medium is drained away. A wound (width 0.5 mm) is created on an area of cells by gentle scraping with a rubber stick and moving back and forth against the top of the culture. The wells are washed four times with PBS to remove remaining cellular debris. Cultures are maintained with a medium supplemented with 5% (FBS). The herbal extract is added to the culture at 100 mg/mL, and the control culture received only PBS. Wound restoration is photographed at 16, 25, and 40 h after injury. And to study the effect of herbal extract on epidermis formation, the keratinocytes are raft cultured, and when the cells reached 90% of confluency, they are trypsinized on a collagen matrix for raft culture as follows. Mouse fibroblasts are mixed with type I collagen matrix at a density of 17,000 cells per Millericell and seeded in 12 mm Millericell. Keratinocytes are then seeded on the matrix at 17,000 cells per Millericell. Cells are cultured submerged in media for 7 d, transferred to the air-liquid interface and then raised for 21 d. The herbal extract is applied to cells with serum-free media at 0, 0.05, 0.5, and 50 mg/mL every 2 d. Part of the epidermal tissue formed by 3 weeks of culture is taken, fixed in Carnoy solution (ethanol-glacial acetic acid-chloroform in 6 : 1 : 3 ratio by volume), washed with 60% then 80% ethanol, and put in paraffin blocks for morphological comparison [27].

## 9. Effect of *In vitro* Studies

The herbal extracts containing compounds with angiogenesis modulating properties showed strong angiogenic activity in CAM treated with herbal extract, by increasing the size and number of blood vessels as compared to control (sterile disc without herbal extract or normal saline solution). For example, the herbal extracts of *Aloe vera* [26], *Alternanthera brasiliana* [25], *Dalbergia odorifera*, *Epimedium sagittatum*, and *Trichosanthes kirilowii* [24] were screened using CAM assay for the analysis of their wound healing potential. The changes in the distribution and density of CAM vessels next to the implant are evaluated by means of a stereomicroscope at regular intervals following the graft procedure, and the percentage increase of blood vessels could be calculated by the following formula:

(1) $$\begin{array}{cc} & \frac{\left(\text{Vessel}\;\text{number}\;\text{of}\;\text{CAM}\;\text{treated}\;\text{by}\;\text{herbal}\;\text{extract}-\text{Vessel}\;\text{number}\;\text{of}\;\text{CAM}\;\text{treated}\;\text{by}\;\text{normal}\;\text{saline}\right)}{\left(\text{Vessel}\;\text{number}\;\text{of}\;\text{CAM}\;\text{treated}\;\text{by}\;\text{normal}\;\text{saline}\right)}\\ & \;\;\times 100\\ & \;=\;\text{Percentage}\;\text{increase}\;\text{of}\;\text{blood}\;\text{vessels}. \end{array}$$

The herbal extract promotes fibroblast proliferation and motility; this activity was also greater than that of a positive control in which fibroblasts were allowed to grow in 10% FCS. The use of 10% FCS to indicate HDF growth stimulation is an established comparative technique and provides a suitable positive control. The herbal extracts of *Celosia argentea* [30], *Buddleja globosa* [31], *Onosma argentatum* [32], and *Scrophularia nodosa* [23] were analysed using fibroblasts bioassay for the determination of their wound healing potential. The activity of herbal extract neither alters motility and proliferation of primary keratinocytes nor show any increase or decrease in EGFR (epidermal growth factor receptor) phosphorylation. It only stimulated the downstream effector in ERK (extracellular signal regulated kinase) activation when compared with control. For example, the herbal extracts of *Chromolaena odorata* [29], *Combretum smeathmanni*, *Phyllanthus muellerianus*, and *Pycnanthus angolensis* [33] were analysed using keratinocytes assay for the determination of their wound healing potential. 

## 10. *In vivo* Studies

The small mammals have emerged as the model of choice for such studies, which are beneficial for multiple reasons. They are inexpensive, easily obtainable, require less space, food, and water, easy to maintain, and can be genetically modified [34]. Additionally, they often have multiple offspring, which develop quickly allowing experiments to proceed through multiple generations. Small animals usually have accelerated modes of healing in comparison to humans, thus experiment duration lasts for days, in contrast to weeks or months in human experiments. Some small mammals can easily be altered genetically and provide a wound model capable of approximating defective human conditions such as diabetes, immunological deficiencies, and obesity [35]. Another advantage of small mammal models is their ability to serve in experiments where death is an endpoint.

## 11. Animal Ethics Approval

Animal Ethics Committee (AEC) is institutionalised in many countries, which regulates and authorises all use of animals for research, teaching, or experimentation subjected to their variable ethical principles and regulatory guidelines. The acquisition and use of animals for research or teaching must not commence before all information requested by the AEC has been supplied and approval has been granted by the AEC. The written proposals should place before the AEC for ethics approval providing sufficient information to satisfy the AEC that the proposed use of animals is justified and complies with the principles of replacement, reduction, and refinement. Adequate care, housing, and handling are maintained. Proper and adequate postprocedural care, including appropriate veterinary attention, must be provided for the animals. Animals must be treated humanely and in accordance with the Act, and regulations and all procedures are to be carried out in accordance with the Code of Practice for the Care and Use of Animals for Scientific Purposes [36].

## 12. Animal Handling

Animal of either sex, same age group, and approximately of similar weight are employed following an acclimatization period of 2–14 d. They are maintained at a well-ventilated animal house under standard controlled conditions at temperature 22 ± 1°C to 30 ± 1°C, relative humidity 35 ± 5 to 65 ± 5%, and kept under either 10/14 or 12/12 h light/dark cycles with free access to food and water *ad libitum*. The animals are housed individually in clean, sterile polyvinyl/propylene/metal cages containing autoclaved paddy husk or paper cuttings as bedding. The animals are fasted/starved for 12–14 h before tests to achieve better drug absorption through the gastrointestinal tract but given unrestricted access to clean drinking water [25].

## 13. Drug Formulation

The next step is to prepare herbal extract formulation (HF) for either oral or topical administration for the treatment of wound. 

### 13.1. Oral Formulation

Herbal extract formulation for oral (HFO) administration is prepared by dissolving 2 g of gum acacia in 100 mL of normal saline. From this, 10 mL of solution, which contains 200 mg of gum acacia, is used for dissolving 1 g of herbal extract. So that each mL of solution contains 100 mg of herbal extract [10]. The dried herbal extract may be dissolved in drinking water or tween-80 (0.5%–1.5%) [37]. Since an average rat consumes 110 mL of water/kg/day, thus 100–500 mg herbal extract can be dissolved in 100 mL of drinking water [38]. In case of aqueous extract, a 20% suspension of the leaves is prepared in 1% gum acacia. The water content of the leaf is found to be about 80%. The aqueous extract is diluted in the proportion of 5 mL of extracts for each 995 mL of distilled water (final concentration = 100 mg/litre). The suspension of herbal extract (5%) can be prepared in gum tragacanth (1%-2% w/v) or in acacia (5%) as suspending/emulsifying agent using distilled water [39]. Aloe vera extracts in the form of suspension (5% acacia) is used as standard. The herbal extract formulations are prepared every fourth day.

### 13.2. Topical Formulation

Herbal extract formulation for topical (HFT) administration is prepared in the form of cream, gel, or ointment according to the choice of application. For the preparation of cream the emulsion of oil and water are added in approximately equal proportions. Oil phase ingredients and water phase ingredients are heated separately in double boiling method at 75°C. At the same temperature, the water phase ingredients are added into oil phase while stirring. Finally, a milky solution is formed. The herbal extract and preservative are added after temperature is reduced to 35°C [40].

The gel is formulated by wetting polymer using deionise water. After the polymer becomes fully wet and the mixture became soggy, it is homogenized using a homogenizer. Without using heat, the mixture is added with neutralizer, triethanolamine, and other ingredients such as glycerine as humectants, moisturizer, chelating agent, and preservative. Lastly, herbal extract diluted with propylene glycol is added [41]. Ointment is a homogeneous, semisolid emollient, formulated using hydrophobic, hydrophilic, or water-emulsifying base to provide preparation that is immiscible, miscible, or emulsifiable with skin secretions. Ointments can be derived from hydrocarbon (fatty), absorption, water-removable, or water-soluble bases. Ointments are prepared by either incorporating the active ingredient(s) into the chosen base or by melting the base and active ingredient(s) together [42].

## 14. Physicopharmaceutical Evaluation

The HFT is evaluated for various physicochemical parameters. Rheological properties like apparent viscosity can be determined using Brookfield viscometer, readability, extrudability, and flow index. Stability is determined by exposing the formulation to various temperatures for a specific period and if no change occurs in the properties, it confirms that the formulation is stable. The pH is determined using a pH meter. External characters such as colour, odour, smoothness, grittiness, and homogeneity are determined by visual inspection and tested for appearance with no lumps. For skin irritation test of each formulation, five human volunteers are selected, and 1 gm of weighed formulation is applied on an area of 2 square inch to the back of their hand and covered with cotton. The volunteers are asked to report after 24 h to observe for any reaction or irritation [40, 42].

## 15. Acute Toxicity and Lethality Test

An acute toxicity study for the herbal extracts in experimental animals is conducted by staircase method. The animals are starved overnight fed orally with the increasing doses (mg or g/kg body weight) of the herbal extract and continuously observed for mortality and behavioural responses for 48 h and thereafter once daily up to 14 d after administration. The 1/10th of the lethal dose is taken as an effective dose ED_50_ (therapeutic dose). The acute toxicity and lethality (LD_50_) of the extract can also be determined using the method of Lorke [43]. Animals in groups received one of 10, 100, or 1000 mg/kg of herbal extract suspended in 3% v/v Tween-85 administered intraperitoneally and observed for 24 h for a number of deaths. From the results of the first test, 200, 400, 800, and 1600 mg/kg doses of the herbal extract are administered to a fresh batch of animals at one animal per dose and the number of deaths in 24 h is recorded. The LD_50_ is calculated as the geometric mean of the highest nonlethal dose (800 mg/kg) and the lowest lethal dose (1000 mg/kg) [20]. The up and down method can also be adopted for acute toxicity studies in which the doses are adjusted by a constant multiplication factor. The dose for each successive animal is adjusted depending on the previous outcome. The therapeutic dose is selected based upon the maximum cutoff value [25].

## 16. Wound Creation

All the surgical interventions are carried out under sterile conditions under general anaesthesia. The predetermined area for wound infliction at the back of the animal is prepared for surgery by removing hairs with depilatory cream/shaving machine/razor. The animal is anaesthetized with anaesthetic ether/chloroform by open mask method or intraperitoneally with anaesthetic drug (35 mg pentobarbitone sodium/25 mg thiopentone sodium/10 mg ketamine/40 mg thiopental/60 mg pentobarbital sodium/0.3 mg chloralhydrate solution per kg body weight of an animal) and placed on the operation table in its natural position. The animal can also be anaesthetized using a combination of anaesthesia (90 mg ketamine + 10 mg xylazine/10 mg xylazine HCl + 50 mg ketamine HCl/50 mg ketamine HCl + 5 mg diazepam/25 mg Ketamine HCl + 5 mg Diazepam per kg body weight of an animal). The induction of localized anesthesia can be done by subcutaneous injection of a lodocain solution (2 mL, 2%) or lignocain HCl (1 mL, 2%) at and around the area under investigation to render area painless. The animals are allowed to recover, housed individually in their cages, and monitored for respiration, colour, and temperature. They are maintained under standard husbandry conditions and on a uniform diet and managed throughout the experimental period. Animals are closely observed for any infection; those who show signs of infection are separated and excluded from the study. They are periodically weighed before and after the experiments. 

### 16.1. Excision Wound Model

Excision wounds are inflicted on the dorsal thoracic region 1–1.5 cm away from the vertebral column on either side and 5 cm away from the ear. After wound area preparation with 70% alcohol, using a sterile round seal of 2.5 cm diameter or a surgical blade or 5–8 mm biopsy punch, the circular skin from the predetermined area on the depilated back of the animal is excised to its full thickness to obtain a wound area of about 200–500 mm^2^ diameter and 2 mm depth. Haemostasis is achieved by blotting the wound with a cotton swab soaked in normal saline [38, 44]. The respective therapeutic treatment is administered either orally or topically to the animals of respective groups until complete epithelialization starting from the day of operation. Collagen estimation, percentage wound contraction, and period of epithelialization parameters are studied.

### 16.2. Incision Wound Model

After wound area preparation with 70% alcohol, two longitudinal paravertebral incisions are made through the skin and cutaneous muscles at a distance of about 1.5 cm from the midline on either depilated side of the vertebral column with a sterile sharp surgical blade. Each incision made is 4–6 cm in length, and after complete haemostasis, the parted skin is stitched with interrupted sutures, 0.5–1.0 cm apart using black braided silk surgical thread (no. 000) and a curved needle (no. 11). The continuous threads on both wound edges are tightened for good closure of the wound. The wounds were left undressed and mopped with a cotton swab [45, 46]. The respective therapeutic treatment is administered either orally or topically to the animals of respective groups until 7th–9th day starting from the day of operation. The sutures were removed on 7th day, and the skin breaking strength of the healed wound is measured on 8th–10th day.

### 16.3. Dead Space Wound Model

In this model, the physical and mechanical changes in the granuloma tissue are studied. The subcutaneous dead space wounds are inflicted one on either side of axilla and groin on the ventral surface of each animal, by making a pouch through a small nick in the skin. The cylindrical grass piths (2.5 × 0.3 cm) or sterile cotton pellets (5–10 mg each) are introduced into the pouch. Each animal received 2 grass piths/cotton pellets in different locations [14, 38]. The dead space wound is created by subcutaneous implantation of a sterilized, shallow, metallic ring (2.5 × 0.3 cm) known as the cylindrical pith or polypropylene tube (2.5 × 0.5 cm) on each side beneath the dorsal paravertebral lumbar skin surface [47, 48], and wounds are sutured. The respective therapeutic treatment is administered either orally or topically to the animals of respective groups for 10 consecutive days. The physical changes in the granuloma tissue are studied in this model.

### 16.4. Burn Wound Model

A special metal plate 2 × 2 cm with holder is heated to 60°C and applied to the dorsal area of the animals for 30 s to induce partial thickness burn wound. Second-degree burns wound can be made by placing the 90°C hot plate on the selected dorsal area of the animal for 10 s. While for full thickness burn wound, the metal plate is heated to 100°C and applied to the dorsal area for 30 s [1, 49]. The animal can also be subjected to rectangular burn wounds (20 × 25 mm^2^) using hot (180°C) brass brick weighing 300 g, which is pressed against the shaved skin for 10 s in the treatment group [50]. A cylindrical metal rod (10 mm diameter) is heated over the open flame for 30 s and pressed to the shaved and disinfected surface for 20 s on selected dorsal area of animal under light anaesthesia [51]. Whereas a partial thickness burns wounds can also be inflicted upon animals starved overnight and under mild anaesthesia, by pouring hot molten wax at 80°C into a metal cylinder with 100–300 mm^2^ circular opening, placed on the back of the animal. On solidification of wax after 8–10 min the metal cylinder with wax adhered to the skin is removed, which left distinctly marked circular burn wound [52]. Animals are placed in individual cages after recovery from anaesthesia. The respective therapeutic treatment is administered either orally or topically to the animals of respective groups until the day of scab falling starting from the day of operation. The parameters studied are percentage wound contraction, hydroxyproline content and epithelialisation time.

## 17. Drug Administration

A route of administration (ROA) in pharmacology and toxicology is the path by which a drug, fluid, poison, or other substance is brought into contact with the body. The goal is to deliver the drug to the target organ or tissue, so it can exert its therapeutic effect. The ROA that is chosen may have a profound effect upon the speed and efficiency with which the drug acts. Treatment of wound includes administration of drugs either locally (topical) or systemically (oral or parenteral).

### 17.1. Topical Administration

Application of HFT to the wound area can be done once, twice, or three times (at *n*-hour intervals) a day at the group-dependent time intervals after cleaning with sterile surgical cotton wool in the experimental group animals (EGA) for a period of *n* days, respectively. The HFT is applied at a dose of 100 mg/kg/day to 500 mg/kg/day with a gauze sponge lasted for 10 min. To prevent the possible thermic effect of the application, the temperature of HFT is kept approximately 37°C. The wound area is then covered with a fresh sterile bandage which is secured by adhesive tape. The commercial Solcoserly jelly or Intrasite gel or any standard drug ointment/cream, containing 0.2% to 1% drug (povidone, gentamicin, mupirocin, sulfathiazole, carboxymethyl cellulose, silver sulphadiazine, chlorhexidine gluconate, povidone-iodine, and nitrofurazone) in the same quantity can be applied daily to wounds of reference group animals (RGA), respectively [53]. 

Control group animals (CGA) are administered with the simple ointment base I.P. or B.P. in the same quantity as a placebo control from zero to the day of complete healing/epithelialization or the postoperative day, whichever is earlier. No treatment serves as a negative group. In case, if the herbal extract is directly used, then it is diluted with isopropyl lauroyl sarcosinate. After the operation, 10 mL of the vehicle (0.2% or 2%) is soaked into filter paper discs and placed on the wound. The filter paper is covered with tape and the tape is covered with film dressing. The sample is reapplied and the wound covered again with film dressing at one-week intervals. Since modern medicine uses antibacterial agents for treatment of wound, the herbal drug aloe vera (10%) in carbopol can be used as standard [54].

### 17.2. Oral Administration

HFO is given to the EGA at a dose of 100 mg/kg/day to 500 mg/kg/day for *n* days in case of incision and dead space wound starting from the day of wound creation. In case of excision wound model and burn wound model, the treatment is continued until the day of scab falling. The CGA received plain drinking water only. The CGA can also be given normal physiological saline or only Tween-80 (0.5%–1%) [37, 47].

## 18. Evaluation of Wound Healing

Wound contraction, which contributes to wound closure, is expressed as a reduction in percentage of the original wound size is studied starting from the day of operation until the day of complete epithelialization and evaluated to calculate the degree of wound healing. Wound tissues are analyzed for hydroxyproline content; the collagen composed of amino acid (hydroxyproline) is the major component of extracellular tissue, which gives strength and support. Breakdown of collagen liberates free hydroxyproline and its peptides. Measurement of hydroxyproline, hence, can be used as a biochemical marker for tissue collagen and an index for collagen turnover. The biochemical marker, hexosamine, a component of the ground substance for the synthesis of the extracellular matrix is evaluated in granulation tissues of excision wounds in order to monitor the wound healing process. Since the level of hexosamine is increased between 7th–12th post wounding day and then decreases slowly; the granulation tissue is obtained from wound area on 11th post wounding day [55, 56].

One of the most crucial phases in dermal wound healing is the progressive increase in biomechanical strength of the tissue; the mechanical properties of the skin are mainly attributed to the function of the dermis in relation to the structure of collagen and elastic fibre networks. Breaking strength of the healed wound is measured as the minimum force required to break the incision apart. Skin breaking strength gives an indication of the tensile strength of wound tissues and represents the degree of wound healing. Tensile strength has commonly been associated with the organization, content, and physical properties of the collagen fibril network. Tensile strength is the resistance to breaking under tension; it indicates how much, the repaired tissue resists breaking under tension and may indicate in part the quality of the repaired tissue [10]. The sutures were removed on the 7th–9th post wounding day, and the tensile strength was measured on the 8th–10th day. The mean tensile strength on the two paravertebral incisions on both sides of the animal is taken as the measures of the tensile strength of the wound for an individual animal [57].

### 18.1. Percentage Wound Contraction

The progressive reduction in the wound area is monitored planimetrically by tracing the raw wound boundaries initially on a sterilized transparency paper sheet in mm^2^ without causing any damage to the wound area, and then, the wound area recorded is measured using a graph paper on every 2–4 d interval. The period of epithelialization is expressed as the number of days required for falling of the eschar (dead-tissue remnants) without any residual raw wound is considered as the end point of complete epithelialisation [58]. Percentage wound contraction is calculated as:

Percentage wound contraction on

(2) $$\begin{array}{cc} N\text{th day}-\text{100} & =\frac{\text{wound area on}\;\;N\text{th day}}{\text{wound area on 1st day}}\times 100. \end{array}$$

### 18.2. Collagen Estimation

On the 11th post wounding day, the animals from each group are euthanized and the wound tissue is excised, weighed, and dried in an oven at 60°C–70°C for 12–18 h, and the dry weight is noted. The tissues are hydrolyzed in 6 N HCl for 24 h at 110°C in sealed glass tubes. The hydrolysate is neutralized to pH 7.0. The sample (200 *μ*L) is mixed with 1 mL of 0.01 M CuSO_4_ followed by the addition of 1 mL of 2.5 N NaOH and then 1 mL of 6% H_2_O_2_. The solution is mixed and shaken occasionally for 5 min. All the tubes are incubated at 80°C for 5 min with frequent vigorous shaking. Upon cooling, 4 mL of 3 N H_2_SO_4_ is added with agitation. Finally, 2 mL of 5% para-dimethyl-aminobenzaldehyde is added to develop a pink colour. The samples are incubated at 70°C for 16 min, cooled by placing the tubes in water at 20°C, and the absorbance is measured at 540 nm using a colorimeter/spectrophotometer. The amount of hydroxyproline in the samples is calculated using a standard curve prepared with pure L-hydroxyproline at the same time [51]. The concentration of the sample is calculated as:

(3) $$\begin{array}{cc} & \text{Concentration}\;\text{of}\;\text{the}\;\text{sample}\\ & =\frac{\text{OD}\;\text{of}\;\text{the}\;\text{sample}}{\text{OD}\;\text{of}\;\text{Standard}}\times \text{concentration}\;\text{of}\;\text{standard}. \end{array}$$

### 18.3. Hexosamine Estimation

The granulation tissue is obtained from wound area on 11th post wounding day is dried in an oven at 60°C and hydrolyzed with 2 N HCl at 100°C for 2 h. The hydrolyzed solution is filtered and pH of the filtrate is adjusted to 6-7. This acid hydrolysate solution is subjected to deamination and nondeamination of hexosamine to determine the amount of hexosamine. For deamination, 0.5 mL of a 5% solution of sodium nitrite and 0.5 mL of a 33% solution of acetic acid is added to 0.5 mL of acid hydrolysate solution. The tubes are shaken and left for 10 min for complete deamination. The excess nitrous acid is removed by adding 0.5 mL of a 12.5% solution of ammonium sulfamate and by shaking the mixture for 30 min. For indole reaction, 2 mL of 5% HCl and 0.2 mL of a 1% solution of indole in alcohol is added to 2 mL of the deaminated hexosamines. The tubes are immersed for 5 min in a boiling water bath. An intense orange colour and a slight turbidity is seen. To remove turbidity, 2 mL of alcohol is added, and tubes are shaken. For nondeamination of hexosamine, 1.5 mL of a mixture of equal volumes of solutions of 5% sodium nitrite, 33% acetic acid and 12.5% ammonium sulfamate are added to 0.5 mL of acid hydrolysate solution. This serves as the control without deamination. The indole reaction is carried out on this mixture as described above. The absorbances of the solutions are determined spectrophotometrically at 492 and 520 nm. The absorbance value for the nondeaminated solutions is subtracted from the corresponding absorbance values for the deaminated unknown and standard solutions of glucosamine hydrochlorides. The increase in the difference in absorbance after the deamination procedure is considered as the measure of the amount of hexosamine. The hexosamine value in *μ*g is computed from the standard curve [56].

### 18.4. Skin Breaking Strength

The anesthetized animal is secured to the table, and a line is drawn on either side of the wound 3 mm away from the suture line. Two allice forceps are firmly applied on to the line facing each other. One of the forceps is supported firmly, whereas the other is connected to a freely suspended light weight metal pan/measuring graduated container through a string run over to a pulley. Weight is added to the pan/water is allowed to flow from the reservoir slowly and continuously into the container. A gradual increase in weight is transmitted to the wound site pulling apart the wound edges. As soon as wound gaping appeared, the addition of weight/water flow is stopped, and the weights added to pan/volume of water collected in the container (approximately equal to its weight) is determined and noted as a measure of breaking strength in grams. Three readings are recorded for a given incision wound, and the procedure is repeated on the contralateral wound. The mean reading for the group is taken as an individual value of breaking strength. The mean value gives the skin breaking strength for a given group [11, 16].

In another approach, the animal is anaesthetized, and healing tissue along with normal skin at the two ends is excised. Strips of 8 mm width and 20 mm length are cut out from the excised tissue, which is loaded between the upper and lower holder of the tensile testing machine in such a way that the effective load bearing size is 8 × 8 mm with the wound remaining in the centre [46]. The total breaking load is measured in Newtons (N), and the tensile strength is calculated as mass in kg by the following equation: Tensile strength = Total breaking load/Cross-sectional area.

### 18.5. Granuloma Studies

The day of the wound creation is considered as day zero. Granulation tissue forms on the dead space wound surrounding the implanted pellets/piths is harvested by careful dissection on the 10th post wounding day under light ether anaesthesia. After noting the wet weight of the piece of granuloma excised, it is dried in an oven at 60°C for 12–24 h to obtain a constant dry weight expressed as mg/100 g body weight. The granuloma tissues are trimmed to obtain the rectangular strip measuring about 15 mm in length and 8 mm width to determine its breaking/tensile strength by continuous water flow technique. The dry granulation tissue is used for the estimation of hexuronic acid, hexosamines, hydroxyproline content, which can be assayed calorimetrically/spectrophotometrically, and a piece of wet granuloma is preserved in 10% formaldehyde for histological studies to evaluate the effect of the herbal extract on collagen formation [2, 14]. Granulation tissue is collected in phosphate-buffered saline (maintained at −64°C) for the estimation of antioxidant enzymes like superoxide dismutase (SOD), catalase, reduced glutathione (GSH), and tissues lipid peroxidation [10].

## 19. Histopathological Studies

The regenerated tissue samples are evaluated for the following histological criteria: the extent of re-epithelialization or ulcus in epidermis, the maturation and organization of the epidermal squamous cells, the thickness of the granular cell layer, the degree of the tissue formation in wounds, and their comparison with the normal tissue part. In addition, angiogenesis, congestion, edema, epithelialization, fibroblasts proliferation, intensity and extent of inflammation (cell infiltration), mononuclear and/or polymorphonuclear cells, necrosis, ulceration, neovascularization, and the pattern of collagen depositions in the dermis are qualitatively evaluated to score the epidermal or dermal remodeling. In case of burn wounds, all the histological criteria are evaluated after 25 d and the last day of the treatment period. For this purpose, tissue samples were taken with a small excision containing part of the wound area.

The cross-sectional full-thickness specimen of granulation tissues is obtained on 10th-11th d or at the end of the experiment from the complete healed wounds of all the four groups of surviving animals is subjected to histological examination. The granulation tissues are separately fixed in 10% neutral phosphate buffered formalin solution for 48 h. The formalin-fixed tissues are dehydrated through graded alcohol series, cleared in xylene and embedded in paraffin wax. Serial sections of 4–7 mm thickness from the paraffin embedded regenerated tissue are cut using a microtome. The sections are processed in alcohol-xylene series and stained with hematoxylin and eosin (HE) or Mallory Azan or toluidine blue (TB) stain and observed for the histological changes under a light microscope and photomicrographs are taken. The number of capillaries is counted in HE stained sections, the collagen area in the granulation tissue is measured in Azan-stained sections and metachromatic stained mast cells are examined in TB stained sections. The number of capillaries is counted in three parts of the granulation tissue (upper, center, and lower). The area of collagen synthesis is derived from the area stained blue by the Azan stain. For better appreciation of collagen deposition Van Geison's (VG) stain is used, which stains the collagen fibres pink, and Masson's trichrome (MT) stain is used that stains the collagen fibres green are performed on paraffin sections, followed by photomicrography. Sections are analyzed and scored as mild (+), moderate (++) and severe (+++) for epidermal or dermal remodeling. The severity of inflammation in healed areas is evaluated by counting the mean number of inflammatory cells infiltration per high field (PHF) (×400 magnification) of three samples of each group [59–61].

## 20. Effects of *In vivo* Studies

In the excision and burn wound model studies the herbal extract showed increased rate of wound contraction and decrease in period of epithelization in experimental group animals (EGA) as compared to control (placebo, negative) group animals (CGA). For example, the herbal extracts of *Acacia nilotica* [40], *Aspilia africana* [20], *Emblica officinalis* [61], *Memecylon umbellatum* [53], and *Rubia cordifolia* [41] were screened using excision wound model whereas the herbal extracts of *Achillea millefolium* [50], *Carica papaya* [51], *Centella asiatica* [49], *Cocos nucifera* [52], and *Crocus sativus* [60] were tested employing burn wound model for the analysis of their wound healing potential. 

In the incision wound model studies, the herbal extract showed significant increase in tensile strength in EGA as compared to CGA. For example, the herbal extracts of *Allamanda cathartica* [55], *Alternanthera brasiliana* [25], *Anogeissus latifolia* [57], *Carallia brachiata* [45], and *Cynodon dactylon* [16] were analysed using incision wound model for the determination of their wound healing potential. In the dead space wound model studies, the herbal extract showed significant increase in collagen deposition showing fewer macrophages and fibroblasts and increase in the dry weight of granulation tissue in EGA as compared to CGA with more aggregation of macrophages with few collagen fibres. For example the herbal extracts of *Allium cepa* [44], *Areca catechu* [48], *Berberis lyceum* [47], *Carapa guainensis* [11], and *Carica papaya* [51] were screened employing dead space wound model for the analysis of their wound healing potential.

Microscopic histopathological examination of the sections prepared from the wounds of EGA, CGA, and RGA exhibited the following characteristics. The regenerated tissue section of CGA showed densely inflamed connective tissue with chronic inflammatory cells between the collagen fibers; this shows incomplete wound healing. The tissue of RGA is composed of dense collagen fibers, fibroblasts with round to oval nuclei and blood vessels, many thin walled blood vessels are present, whereas the regenerated tissue section of herbal extract-treated EGA showed fibrous connective tissue with scattered inflammatory cells and fibroblasts. There was a progressive collagenation with few thin-walled blood vessels with small lumina, epithelialisation of tissues was also observed. The EGA showed dense fibrous tissue with thick collagen bundles, fibroblasts, and scattered inflammatory cells. The appearance was almost identical to that of normal tissues. The results showed that the wound healing was faster in the EGA as compared to CGA and RGA [16, 58].

## 21. Discussion

There is an increasing interest in finding herbal extracts with wound healing efficacy although the use of such extracts for treating cuts and wounds is a common practice in traditional medicine. Wound infection resulting from the impaired immunity and exposure or poor hygiene is one of the most commonly encountered and clinically important impediments to wound healing. The injured skin remains vulnerable to invasive microbial infections of all kinds subsequent development of wound sepsis until complete epithelial repairs has occurred [21]. Injury becomes infected, because the wound area is an ideal medium for the multiplication of the infecting organism. Topical antimicrobial therapy is one of the most important methods of wound care [12]. The herbal extracts and fractions effectively arrested bleeding from fresh wounds, inhibited microbial growth and accelerated wound healing [20]. The enhanced wound healing potency of various herbal extracts may be attributed to free radical-scavenging action and the antimicrobial property of the phytoconstituents present in the extract, and the quicker process of wound healing could be a function of either the individual or the synergistic effects of bioactive molecules. These active constituents promote the process of wound healing by increasing the viability of collagen fibrils, by increasing the strength of collagen fibers either by increasing the circulation or by preventing the cell damage or by promoting the DNA synthesis [62].

Pronounced effects on fibroblast motility and cellular proliferation due to the mitogenic activity of herbal extract correlate with its wound healing effect being predominantly dermal. During wound repair, fibroblasts migrate from the wound edges to the wound site, proliferate, and subsequently produce collagen, the main component in the extracellular matrix. Stimulation of fibroblasts is one mechanism by which herbal extracts might enhance the wound repair process. Although keratinocytes also need to migrate from the wound edge to provide a provisional matrix for the fibroblasts to migrate on, this might be accelerated secondary to a mature dermal matrix. These latter effects on fibroblasts probably are the result of phytoconstituents of herbal extract that may have a growth factor-like activity or have the ability to stimulate the early expression of growth factors [30]. Keratinocyte migration is an event in the wound healing process which may sometimes be arrested even when there is a development of good granulation tissue. Migration is influenced by the nature of surrounding extra matrix and the protein composition of the wound environment [29].

It is known that herbal extracts may be contaminated by endotoxins, depending on the conditions and method of preparation. These are lipopolysaccharides (LPS) associated with the cell wall of gram-negative bacteria and have various biological effects. Therefore, the herbal extracts must be assayed for endotoxin and also investigate the effect of an LPS from *E. coli* on the growth of skin cells *in vitro* [29]. Due to increasing evidence that bacteria in wounds with potential chronicity live within biofilm communities, protecting them from host defences, the study of biofilm has become an area of intense scientific investigation in wound healing and bacterial resistance, both of which are relevant to the diabetic state [38].

The increasing use of traditional medicines and therapies demands more scientifically sound evidence for the principles behind therapies and for effectiveness of medicines. Recent advancements in the analytical and biological sciences, along with innovations in genomics and proteomics can play an important role in the validation of these therapies. Modern therapy requires the targeting of drugs directly to the site of interest and to accomplish that goal in systemic treatment. Gene and stem-cell therapy are emerging as a new and promising approach for enhancing treatment of wound healing [63]. 

Embryonic and adult stem cells have a prolonged self-renewal capacity with the ability to differentiate into various tissue types. Stem cells are attracted to areas of wound healing and have the ability to multiply and then divide into the cells needed to repair the damage. Stem cells have revolutionary clinical potential, because they can be used as a therapeutic intervention for treating many injuries, including brain repair and regeneration, in many instances. A variety of sources, such as bone marrow, peripheral blood, umbilical cord blood, adipose tissue, skin and hair follicles, have been utilized to isolate stem cells to accelerate the healing response of acute and chronic wounds [64]. Stem cells harboured within adult bone marrow contribute to wound angiogenesis. These cells, known as endothelial progenitor cells (EPCs), can also be isolated in small numbers from the peripheral circulation of normal healthy adults. Following injury, EPCs are mobilized into the circulation, and they home sites of neovascularization, where they differentiate into adult endothelial cells. Placental growth factor (PlGF), a member of the vascular endothelial growth factor (VEGF) family, and its receptor flt-1 (VEGF-R1) have been identified as regulators for EPC recruitment in angiogenesis. Stem cells could also possibly be an important factor in delaying, slowing, and in some instances, halting, deterioration, even reversing the course of a sudden acute injury or acute onset crisis situation. Enhanced understanding of these cells may help develop novel therapies for difficult cutaneous conditions such as nonhealing chronic wounds and hypertrophic scarring as well as engineering cutaneous substitutes [65]. 

In order to accelerate wound closure, genes encoding for growth factors or cytokines showed the greatest potential. Modern functional genomics approaches may facilitate a better understanding of the molecular events involved in tissue morphogenesis and allow the identification of molecular signatures and pathways. The tools of genomics and proteomics will be used to profile individual wounds in order to tailor therapy for patients. Gene therapy or cell-based therapy delivering angiogenic growth factors and recombinant protein therapy may offer advantages over other wound healing therapies [66]. Novel growth factor delivery systems are now under investigation, such as angiogenic gene sutures, autologous stem-cell transplantation, genetically modified tissue engineered constructs, and growth factor impregnated dressings or sprays. As wound research and technology development continue, these efforts will undoubtedly reveal new opportunities for accelerating wound healing. The functional genomics and proteomics and knowledge of cell signalling networks may contribute substantially to developing a molecular evidence-based CAM and pave the way to novel approaches in tissue engineering and regeneration [67]. 

Novel delivery vehicles, generated through nanotechnology is raising the novel and stirring prospect for controlled and sustained drug delivery across the impenetrable skin barrier is a magnificent clinical application of nanotechnology for wound management. Particles of 500 nm size and smaller exhibit a host of unique properties that are superior to their bulk material counterparts. Small size is a necessary feature, but other properties are needed for nanomaterials to achieve efficacy as a topical delivery vehicle [68]. Additionally, these products should be able to adjust to relevant physiologic variations as part of their design and targeting. The formation of nanocrystalline silver particles that are only several nanometers in diameter greatly increases the available surface area compared to bulk silver and dramatically changes its properties. Silver nanoparticles have been shown to have many beneficial properties for wound management, including antibacterial activity, antifungal activity, and even anti-inflammatory properties [69]. 

Nanofibers also exhibit properties that may be beneficial in burn care, including a large surface area to volume ratio, high porosity, improved cell adherence, and controlled *in vivo* degradation rates. Thus, the nanofiber scaffolds have immediate applications as dressings for burn wounds and significant potential in drug delivery [70]. The delivery of nitric oxide (NO) radicals using nanotechnology is possible by trapping NO radicals stably within a dry matrix, from where they can be released over an extended period and at a relatively fixed concentration. The potential of NO-nanoparticles in wound management has been demonstrated in numerous animal studies, suggesting that this technology may ultimately offer an effective alternative in the treatment of wound infections [71]. Recombinant human epidermal growth factor (rhEGF) nanoparticles is prepared by utilizing a modified double-emulsion method with poly(lactic-co-glycolic acid), as the carrier and the controlled release of rhEGF nanoparticles enhances its effects in stimulating proliferation of the mouse fibroblasts and shorten the wound healing time in diabetic rats with full-thickness wounds, divided into four groups according to treatments: rhEGF nanoparticles, rhEGF stock solution, empty nanoparticles, and phosphate-buffered saline [72].

The complex cellular and molecular signalling and regulatory processes underlying development and homeostasis can only be understood comprehensively by analysis in the intact living organism. Molecular interactions are frequently transient and context dependent, necessitating *in vivo* analysis to generate relevant insights into the molecular mechanisms. Biomedical research and drug development have thus an increasing need to analyse and monitor these dynamic processes in cellular physiology, development, and disease in the living organism using bioimaging technologies [73]. Bioimaging enables the discovery and tracing of biomarkers of drug response is an increasingly important field of advanced research. Biomarkers are biological indicators of disease or healing effects that can be measured by *in vivo* biomedical imaging and molecular imaging in particular, as well as other *in vitro* or laboratory methods. Recent work has shown that biomedical imaging can provide an early indication of drug response by use of X-ray, CT, or PET-CT. Bioimaging will help scientists to probe the complex relationships between genes, proteins, cellular components, and physiological systems. Nano-bio-imaging is the entirety of various kinds of imaging technology supported by nanotechnology, and it enables further progress of research in nanomedicine and nanotechnology. This new form of bioimaging will play a powerful role in the emerging field of systems biology.

These technologies allow precise tracking of metabolites which can, in turn, be used as biomarkers for disease identification, progress, and treatment response. Discovery and development of disease biomarkers in animal models can then be transferred to use in clinical settings. In the realm of nanotechnology, drug delivery to specific targets, controlled drug release and monitoring offers opportunities for molecular imaging technologies to make significant impacts on both pharmacology and early clinical trials [74]. The *in vitro* assays are relatively inexpensive and give more rapid results for initial screening of large numbers of agents. Definitive conclusions cannot be based on *in vitro* assays alone. However, an ability to inhibit endothelial cell proliferation, migration and tubule formation *in vitro* may not predict *in vivo* response. *In vivo* assays provide a more complete physiologic assessment of angiogenesis, but they are more time consuming and expensive.

Wound healing generally requires support at three levels. First, improving general resistance and support mechanisms that could be obtained from rejuvenative, adaptogenic, palliative, antioxidant, cleansing, detoxifying, buffering, and lubricous activities. Second, stimulating the repair and regenerative mechanisms to prolong cell life, cell migration and cell binding, remove skin blemishes, and improve tensile strength or elasticity of the skin, improve moisture-holding capacity of skin. Third, therapeutic and nutritional activities including anti-inflammatory, antiseptic, and antimicrobial, protein and collagen synthesis and increased stability of biomembranes. Antioxidants can interfere with the oxidation process by reacting with free radicals, chelating catalytic metals and by acting as oxygen scavengers. Free radicals and other reactive oxygen species (ROSs) are considered to be important causative factors in delaying the healing process [75]. 

At high concentrations, ROSs can induce severe tissue damage and even lead to neoplastic transformation, which further impede the healing process by causing damage to cellular membranes, DNA, proteins, and lipids as well. Hence, if an herbal extract having antioxidant potentials and an additional antimicrobial activity, it could be a good therapeutic agent for accelerating the wound healing process [76]. Oxidative stress also plays an important role in impaired wound healing. This oxidative stress may cause damage to the growing tissue at the repair site. In case of burn wound, microcirculation is generally compromised, resulting in a restriction or cessation of blood flow, which in turn causes ischemia and subsequently a reperfusion, and thus generates oxidative stress. Intervention that could restore blood flow or scavenging the free radicals should, in principle, reduce the damage due to oxidative stress [49]. Thus, the antioxidants present in the herbal extract could be expected to promote epithelization by controlling oxidative stress. Botanicals with antioxidant or free radical-scavenging activity thus can play a significant role in healing of wounds [39]. 

Pharmaceutically as well as biologically, the herbal extracts promote fast wound healing than control and non medicated group in different *in vivo* studies. The process of wound healing is promoted by several herbal extracts, which are composed of active agents like triterpenes, alkaloids, flavonoids, tannins, saponins, anthraquinones, and other biomolecules [56, 61]. A number of secondary metabolites/compounds isolated from plants have been demonstrated in animal models as active principles responsible for facilitating healing of wounds. Some of the most important ones include oleanolic acid, polysaccharides, gentiopicroside, sweroside, swertiamarin, shikonin derivatives (deoxyshikonin, acetyl shikonin, 3-hydroxy-isovaleryl shikonin, and 5,8-Odimethyl acetyl shikonin), asiaticoside, asiatic acid, madecassic, quercetin, isorhamnetin, kaempferol, curcumin, sesamol (3,4 methylenedioxyphenol), coluteol, colutequinone B, hyperforin, catechins, and isoflavonoids that could potentially be new therapeutic agents to treat wounds. These agents usually influence one or more phases of the healing process [41]. 

Tannins act as free radical scavengers, triterpenoids, and flavonoids promote wound healing due to their astringent and antimicrobial property, and saponins due to their antioxidant and antimicrobial activity, which appear to be responsible for wound contraction and elevated rate of epithelialization. Flavonoids also possess potent antioxidant and free radical-scavenging effect, enhancing the level of antioxidant enzymes in granuloma tissue [44]. Sterols and polyphenols are also responsible for wound healing due to free radical-scavenging and antioxidant activity, which are known to reduce lipid peroxidation, thereby reduce cell necrosis and improving vascularity [40]. Several types of injuries like burn, wounds, and skin ulcers usually generate superoxides and lipid peroxidation through the activation of neutrophils. Hence, any drug that inhibits lipid peroxidation is believed to increase the viability of collagen fibrils by increasing the strength of collagen fibres, increasing the circulation, preventing the cell damage, and by promoting the DNA synthesis. Better collagenation seen under the influence of some herbal extracts may be because of improved antioxidant status. Thus, an intervention into any one of these phases by drugs could eventually lead to either promotion or depression of the collagenation phase of healing [77]. 

**Figure 4:**
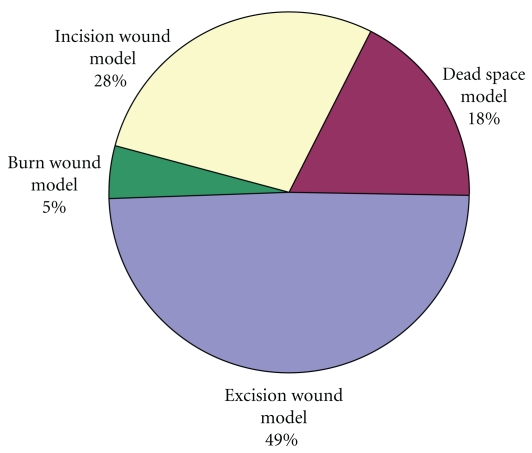
Percentage utilization of different wound models for *in vivo* studies, calculated from the published research articles.

The need for *in vivo* studies arose from the fact that the wound healing process involves granulation, collagenation, collagen maturation and scar maturation are some of many phases and very few drugs are available that can act directly on all the phases. Whereas the use of a single model is inadequate and there is no reference standard, which can collectively represent the various components of wound healing, as drugs which influence one phase may not necessarily influence another. Hence, different models are necessary to assess the effect of herbal extract on various phases of wound healing, which run concurrently, but independent of each other [37]. Percentage utilization of different wound models for *in vivo* studies is presented in Figure 4. The data was calculated from the published original research articles compiled from various sources during the composition of this paper. 

Topically administered drugs are effective in faster wound contraction due to the larger availability at the wound site. An ointment with water-soluble base is of first choice due to their ease of preparation and eases of cleaning after application. The medicaments are dispersed in the base, and later they get divided after the drug penetration into the living cells of skin [78]. The results of various studies performed so far are significant for different parameters in wound healing activity when compared with the control group and showed that the usage of herbal extracts significantly accelerated wound healing. Increased hydroxyproline content is a reflection of increased collagen content, which implies an effect on the early granulation or proliferation phase in treated wounds. Increased hexosamine content reflects the stabilization of collagen molecules by enhancing electrostatic and ionic interactions with it, which in turn reflects remodeling of the new extracellular matrix produced [30]. 

Essential trace elements especially zinc and vitamin C, vitamin E also influences the process of wound repair. Zinc is one of the essential trace elements and serves as a cofactor in various enzyme systems, including zinc-dependent matrix metalloproteinases, which augment autodebridement and keratinocyte migration during wound repair processes. It also provides resistance to epithelial apoptosis via cytoprotection, probably through antioxidant activity of the cysteine-rich metallothioneins, against reactive oxygen species and bacterial toxins. Deficiency of zinc in the body, either hereditary or dietary, can lead to delayed wound healing. Zinc content of the plant extracts used for wound healing purposes might have a great contribution in the healing process. Topical administration of zinc is superior to oral administration because of its effect in reducing super infections and necrosis via enhanced local defence systems and the sustained release of zinc ions, which stimulates re-epithelialization of wounds. Vitamin C also acts as cofactors or coenzymes in a number of metabolic functions involved in wound healing. Hence, zinc and vitamin C levels of the herbal extracts can be determined [79].

**Figure 5:**
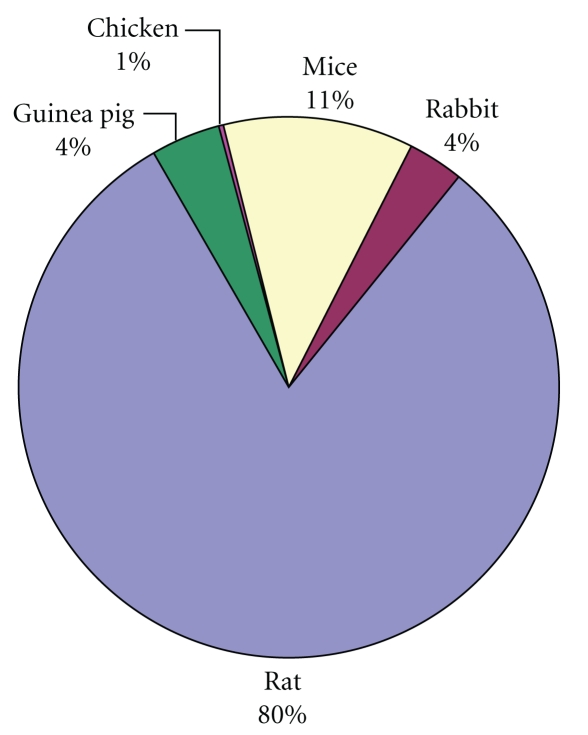
Percentage utilization of different animals for *in vivo* studies, calculated from the published research articles.

When attempting to use or develop a model for wound healing studies, the benefits and disadvantages must be considered carefully, since a model attempts to imitate some other situation. Generally, animal models approximate human wound healing studies better than *in vitro* assays. However, the choice of animal can be difficult, since its ability to represent human wound healing must be coupled with its practicality. Percentage utilization of different animals for *in vivo* studies is presented in Figure 5, the data of which was calculated from the published original research articles. Although the rat, mouse, rabbit, and guinea pigs wound models exist, swine skin is the most similar to humans and has been shown to be an excellent tool to evaluate wound healing therapies. Porcine skin is structurally similar to human skin with similar epidermal thickness and dermal-epidermal thickness ratios. The hairless mouse can be selected as a wound model for epidermal injury, because it offers several advantages. First, the epidermis does not have a fur coat, which interferes with the separation of epidermis from dermis; second, the size and economy of a hairless mouse make it an ideal model to evaluate the effects of pharmacological agents on the wound healing process [27].

## References

[1] Shuid AN, Anwar MS, Yusof AA (2005). The effects of *Carica papaya* Linn. latex on the healing of burn wounds in rats. *Malaysian Journal of Medicine and Health Sciences*.

[2] Jalalpure SS, Agrawal N, Patil MB, Chimkode R, Tripathi A (2008). Antimicrobial and wound healing activities of leaves of *Alternanthera sessilis* Linn. *International Journal of Green Pharmacy*.

[3] Martin P (1997). Wound healing—aiming for perfect skin regeneration. *Science*.

[4] Nayak BS, Pinto Pereira LM (2006). *Catharanthus roseus* flower extract has wound-healing activity in Sprague Dawley rats. *BMC Complementary and Alternative Medicine*.

[5] Hwang JK, Kong TW, Baek NI, Pyun YR (2000). *α*-Glycosidase inhibitory activity of hexagalloylglucose from the galls of *Quercus infectoria*. *Planta Medica*.

[6] Akinmoladun AC, Ibukun EO, Afor E (2007). Chemical constituents and antioxidant activity of *Alstonia boonei*. *African Journal of Biotechnology*.

[7] Edeoga HO, Okwu DE, Mbaebie BO (2005). Phytochemical constituents of some Nigerian medicinal plants. *African Journal of Biotechnology*.

[8] Kosger HH, Ozturk M, Sokmen A, Bulut E, Ay S (2009). Wound healing effects of *Arnebia densiflora* root extracts on rat palatal mucosa. *European Journal of Dentistry*.

[9] Handa SS, Handa SS, Khanuja SPS, Longo G, Rakesh DD (2008). An overview of extraction techniques for medicinal and aromatic plants. *Extraction Technologies for Medicinal and Aromatic Plants*.

[10] Shetty S, Udupa S, Udupa L (2008). Evaluation of antioxidant and wound healing effects of alcoholic and aqueous extract of *Ocimum sanctum* Linn in rats. *Evidence-Based Complementary and Alternative Medicine*.

[11] Nayak SB, Kanhai J, Milne DM, Pereira LP, Swanston WH (2011). Experimental evaluation of ethanolic extract of *Carapa guianensis* L. leaf for its wound healing activity using three wound models. *Evidence-Based Complementary and Alternative Medicine*.

[12] Esimone CO, Nworu CS, Jackson CL (2009). Cutaneous wound healing activity of a herbal ointment containing the leaf extract of *Jatropha curcas L.* (Euphorbiaceae). *International Journal of Applied Research in Natural Products*.

[13] Vimal A, Suseela L, Vadivu R (2009). Wound healing activity of ethanolic extract of aerial parts of *Datura fastuosa* Linn on Wistar albino rats. *Journal of Pharmacy Research*.

[14] Patil DN, Kulkarni AR, Shahapurkar AA, Hatappakki BC (2009). Natural cumin seeds for wound healing activity in albino rats. *International Journal of Biological Chemistry*.

[15] Orafidiya LO, Fakoya FA, Agbani EO, Iwalewa EO (2005). Vascular permeability increasing effect of the leaf essential oil of *Ocimum gratissimum* Linn as a mechanism for its wound healing property. *The African Journal of Traditional, Complementary and Alternative Medicines*.

[16] Garg VK, Khosa RL, Paliwal SK (2009). Wound healing activity of aqueous extract of *Cynodon dactylon*. *Pharmacologyonline*.

[17] Mustafa MR, Mahmood AA, Sidik K, Noor SM (2005). Evaluation of wound healing potential of *Ageratum conyzoides* leaf extract in combination with honey in rats as animal model. *International Journal of Molecular Medicine and Advance Sciences*.

[18] Wagner H, Bladt S, Zgainski EM (1984). *Plant Drug Analysis*.

[19] Harborne JB (1998). *Phytochemical Methods: A guide to Modern Techniques of Plant Analysis*.

[20] Okoli CO, Akah PA, Okoli AS (2007). Potentials of leaves of *Aspilia africana* (Compositae) in wound care: an experimental evaluation. *BMC Complementary and Alternative Medicine*.

[21] Odimegwu DC, Ibezim EC, Esimone CO, Nworu CS, Okoye FBC (2008). Wound healing and antibacterial activities of the extract of *Dissotis theifolia* (Melastomataceae) stem formulated in a simple ointment base. *Journal of Medicinal Plants Research*.

[22] Margaret I, Srinivasa RP, Kaiser J (1998). Antiinflammatory profile of *Tridax procumbens* in animal and fibroblast cell models. *Phytotherapy Research*.

[23] Stevenson PC, Simmonds MSJ, Sampson J, Houghton PJ, Grice P (2002). Wound healing activity of acylated iridoid glycosides from *Scrophularia nodosa*. *Phytotherapy Research*.

[24] Wang S, Zheng Z, Weng Y (2004). Angiogenesis and anti-angiogenesis activity of Chinese medicinal herbal extracts. *Life Sciences*.

[25] Barua CC, Talukdar A, Begum SA (2009). Wound healing activity of methanolic extract of leaves of *Alternanthera brasiliana* Kuntz using *in vivo* and *in vitro* model. *Indian Journal of Experimental Biology*.

[26] Gupta A, Upadhyay NK, Sawhney RC, Kumar R (2008). A poly-herbal formulation accelerates normal and impaired diabetic wound healing. *Wound Repair and Regeneration*.

[27] Choi SW, Son BW, Son YS, Park YI, Lee SK, Chung MH (2001). The wound-healing effect of a glycoprotein fraction isolated from *Aloe vera*. *British Journal of Dermatology*.

[28] Gál P, Toporcer T, Grendel T (2009). Effect of *Atropa belladonna* L. on skin wound healing: biomechanical and histological study in rats and in vitro study in keratinocytes, 3T3 fibroblasts, and human umbilical vein endothelial cells. *Wound Repair and Regeneration*.

[29] Phan TT, Hughes MA, Cherry GW (2001). Effects of an aqueous extract from the leaves of *Chromolaena odorata* (Eupolin) on the proliferation of human keratinocytes and on their migration in an in vitro model of reepithelialization. *Wound Repair and Regeneration*.

[30] Priya KS, Arumugam G, Rathinam B, Wells A, Babu M (2004). *Celosia argentea* Linn. leaf extract improves wound healing in a rat burn wound model. *Wound Repair and Regeneration*.

[31] Houghton PJ, Hylands PJ, Mensah AY, Hensel A, Deters AM (2005). In vitro tests and ethnopharmacological investigations: wound healing as an example. *Journal of Ethnopharmacology*.

[32] Ozgen U, Ikbal M, Hacimuftuoglu A (2006). Fibroblast growth stimulation by extracts and compounds of *Onosma argentatum* roots. *Journal of Ethnopharmacology*.

[33] Agyare C, Asase A, Lechtenberg M, Niehues M, Deters A, Hensel A (2009). An ethnopharmacological survey and in vitro confirmation of ethnopharmacological use of medicinal plants used for wound healing in Bosomtwi-Atwima-Kwanwoma area, Ghana. *Journal of Ethnopharmacology*.

[34] Sullivan TP, Eaglstein WH, Davis SC, Mertz P (2001). The pig as a model for human wound healing. *Wound Repair and Regeneration*.

[35] Davidson JM (1998). Animal models for wound repair. *Archives of Dermatological Research*.

[36] Tremayne-Lloyd T, Srebrolow G (2007). Research ethics approval for human and animal experimentation: consequences of failing to obtain approval—including legal and professional liability. *The Journal of the Canadian Chiropractic Association*.

[37] Roshan S, Sadath A, Khan A, Tazneem B, Purohit MG (2008). Wound healing activity of *Abuliton indicum*. *Pharmacognosy magazine*.

[38] Nayak SB, Pereira LP, Maharaj D (2007). Wound healing activity of *Carica papaya* L. in experimentally induced diabetic rats. *Indian Journal of Experimental Biology*.

[39] Kamath JV, Rana AC, Chowdhury AR (2003). Pro-healing effect of *Cinnamomum zeylanicum* bark. *Phytotherapy Research*.

[40] Baravkar AA, Kale RN, Patil RN, Sawant SD (2008). Pharmaceutical and biological evaluation of formulated cream of methanolic extract of *Acacia nilotica* leaves. *Research Journal of Pharmacy and Technology*.

[41] Karodi R, Jadhav M, Rub R, Bafna A (2009). Evaluation of the wound healing activity of a crude extract of *Rubia cordifolia* L. (Indian madder) in mice. *International Journal of Applied Research in Natural Products*.

[42] Jha RK, Garud N, Nema RK (2009). Excision and incision wound healing activity of flower head alcoholic extract of *Sphaeranthus indicus* Linn. in albino rats. *Global Journal of Pharmacology*.

[43] Lorke D (1983). A new approach to practical acute toxicity testing. *Archives of Toxicology*.

[44] Shenoy C, Patil MB, Kumar R, Patil S (2009). Preliminary phytochemical investigation and wound healing activity of *Allium cepa* Linn (Liliaceae). *International Journal of Pharmacy and Pharmaceutical Sciences*.

[45] Krishnaveni B, Neeharika V, Venkatesh S, Padmavathy R, Reddy BM (2009). Wound healing activity of *Carallia brachiata* bark. *Indian Journal of Pharmaceutical Sciences*.

[46] Perez GRM, Vargas SR, Ortiz HYD (2005). Wound healing properties of *Hylocereus undatus* on diabetic rats. *Phytotherapy Research*.

[47] Asif A, Kakub G, Mehmood S, Khunum R, Gulfraz M (2007). Wound healing activity of root extracts of *Berberis lyceum* royle in rats. *Phytotherapy Research*.

[48] Azeez S, Amudhan S, Adiga S, Rao N, Rao N, Udupa LA (2007). Wound healing profile of *Areca catechu* extracts on different wound models in wistar rats. *Kuwait Medical Journal*.

[49] Wannarat K, Tantisira MH, Tantisira B (2009). Wound healing effects of a standardized extract of *Centella asiatica* ECa 233 on burn wound in rats. *Thailand Journal of Pharmacology*.

[50] Tajik H, Jalali FSS (2007). Influence of aqueous extract of Yarrow on healing process of experimental burn wound in rabbit: clinical and microbiological study. *Journal of Animal and Veterinary Advances*.

[51] Gurung S, Škalko-Basnet N (2009). Wound healing properties of *Carica papaya* latex: *in vivo* evaluation in mice burn model. *Journal of Ethnopharmacology*.

[52] Srivastava P, Durgaprasad S (2008). Burn wound healing property of *Cocos nucifera*: an appraisal. *Indian Journal of Pharmacology*.

[53] Puratchikody A, Nagalakshmi G (2007). Wound healing activity of *Memecylon umbellatum* Burm. *Journal of Plant Sciences*.

[54] Kiran K, Asad M (2008). Wound healing activity of *Sesamum indicum* L seed and oil in rats. *Indian Journal of Experimental Biology*.

[55] Nayak S, Nalabothu P, Sandiford S, Bhogadi V, Adogwa A (2006). Evaluation of wound healing activity of *Allamanda cathartica. L.* and *Laurus nobilis. L.* extracts on rats. *BMC Complementary and Alternative Medicine*.

[56] Chaudhari M, Mengi S (2006). Evaluation of phytoconstituents of *Terminalia arjuna* for wound healing activity in rats. *Phytotherapy Research*.

[57] Govindarajan R, Vijayakumar M, Rao CV, Shirwaikar A, Mehrotra S, Pushpangadan P (2004). Healing potential of *Anogeissus latifolia* for dermal wounds in rats. *Acta Pharmaceutica*.

[58] Bhat RS, Shankrappa J, Shivakumar HG (2007). Formulation and evaluation of polyherbal wound treatments. *Asian Journal of Pharmaceutical Sciences*.

[59] Fujita N, Sakaguchi I, Kobayashi H (2003). An extract of the root of *Lithospermun erythrorhison* accelerates wound healing in diabetic mice. *Biological and Pharmaceutical Bulletin*.

[60] Khorasani G, Hosseinimehr SJ, Zamani P, Ghasemi M, Ahmadi A (2008). The effect of saffron (*Crocus sativus*) extract for healing of second-degree burn wounds in rats. *Keio Journal of Medicine*.

[61] Sumitra M, Manikandan P, Gayathri VS, Mahendran P, Suguna L (2009). *Emblica officinalis* exerts wound healing action through up-regulation of collagen and extracellular signal-regulated kinases (ERK1/2). *Wound Repair and Regeneration*.

[62] Majumdar M, Nayeem N, Kamath JV, Asad M (2007). Evaluation of *Tectona grandis* leaves for wound healing activity. *Pakistan Journal of Pharmaceutical Sciences*.

[63] Patwardhan B, Warude D, Pushpangadan P, Bhatt N (2005). Ayurveda and traditional Chinese medicine: a comparative overview. *Evidence-Based Complementary and Alternative Medicine*.

[64] Branski LK, Gauglitz GG, Herndon DN, Jeschke MG (2009). A review of gene and stem cell therapy in cutaneous wound healing. *Burns*.

[65] Wu Y, Wang J, Scott PG, Tredget EE (2007). Bone marrow-derived stem cells in wound healing: a review. *Wound Repair and Regeneration*.

[66] Soulet F, Kilarski WW, Antczak P (2010). Gene signatures in wound tissue as evidenced by molecular profiling in the chick embryo model. *BMC Genomics*.

[67] Cooper EL (2009). Integrative genomics and fecundity. *Evidence-Based Complementary and Alternative Medicine*.

[68] Nasir A (2010). Nanotechnology and dermatology: part I—potential of nanotechnology. *Clinics in Dermatology*.

[69] Tian J, Wong KKY, Ho CM (2007). Topical delivery of silver nanoparticles promotes wound healing. *ChemMedChem*.

[70] Atiyeh BS, Costagliola M, Hayek SN, Dibo SA (2007). Effect of silver on burn wound infection control and healing: review of the literature. *Burns*.

[71] Cevc G, Vierl U (2010). Nanotechnology and the transdermal route. A state of the art review and critical appraisal. *Journal of Controlled Release*.

[72] Chu Y, Yu D, Wang P, Xu J, Li D, Ding M (2010). Nanotechnology promotes the full-thickness diabetic wound healing effect of recombinant human epidermal growth factor in diabetic rats. *Wound Repair and Regeneration*.

[73] Inoue H, Murakami T, Ajiki T, Hara M, Hoshino Y, Kobayashi E (2008). Bioimaging assessment and effect of skin wound healing using bone-marrow-derived mesenchymal stromal cells with the artificial dermis in diabetic rats. *Journal of Biomedical Optics*.

[74] Farokhzad OC (2008). Nanotechnology for drug delivery: the perfect partnership. *Expert Opinion on Drug Delivery*.

[75] Datta HS, Mitra SK, Patwardhan B Wound healing activity of topical application forms based on ayurveda.

[76] Akkol EK, Koca U, Pesin I, Yilmazer D Evaluation of the wound healing potential of *Achillea biebersteinii* Afan. (Asteraceae) by *in vivo* excision and incision models.

[77] Umadevi S, Mohanta GP, Kalaichelvan VK, Manavalan R (2006). Studies on wound healing effect of *Flaveria trinervia* leaf in mice. *Indian Journal of Pharmaceutical Sciences*.

[78] Jain S, Jain N, Tiwari A, Balekar N, Jain DK (2009). Simple evaluation of wound healing activity of polyherbal formulation of roots of *Ageratum conyzoides* Linn. *Asian Journal of Research in Chemistry*.

[79] Suntar IP, Koca U, Akkol EK, Yilmazer D, Alper M (2011). Assessment of wound healing activity of the aqueous extracts of *Colutea cilicica* Boiss. & Bal. fruits and leaves. *Evidence-Based Complementary and Alternative Medicine*.

